# Calreticulin as a prognostic biomarker and correlated with immune infiltrate in kidney renal clear cell carcinoma

**DOI:** 10.3389/fgene.2022.909556

**Published:** 2022-10-21

**Authors:** Ming Sun, Shanshan Qi, Min Wu, Wei Xia, Hao Xiong

**Affiliations:** Department of Hematology and Oncology, Wuhan Children’s Hospital, Tongji Medical College, HUST, Wuhan, China

**Keywords:** CALR, prognostic value, immune infiltration, immunotherapy, renal clear cell carcinoma

## Abstract

**Background:** Calreticulin (CALR) has been investigated in several malignant diseases and is associated with immune-cell infiltration. However, the prognostic value of CALR in kidney renal clear cell carcinoma (KIRC) is still unknown.

**Methods:** Based on the computational analysis, data from 530 KIRC cases and 72 normal kidney samples from The Cancer Genome Atlas (TGCA-KIRC) database were analyzed in this study. The expression of CALR mRNA in pan-cancer and immune infiltrates was analyzed using the Tumor Immune Estimation Resource (TIMER) database. The CALR protein expression was obtained from the UALCAN and Human Protein Atlas (HPA) databases. Survival, functional, and statistical analyses were conducted using *R* software.

**Results:** The CALR expression was higher in KIRC cases than in normal kidneys. A high CALR expression was correlated with TNM stage, pathological stage, and histological grade. Kaplan–Meier survival analysis showed that a high CALR expression was associated with poor overall survival, disease-specific survival, and progression-free interval. Gene set enrichment analysis (GSEA) indicated that CALR was enriched in IL-6 and IL-2 signaling, interferon signaling, TNF signaling, inflammatory response, apoptosis, and the p53 pathway. CALR is correlated with immune-infiltrating cells. A significant correlation was observed between CALR expression and immunomodulators.

**Conclusion:** We identified CALR as a prognostic biomarker of KIRC. Meanwhile, the CALR expression associated with immune infiltration indicated that CALR might be a potential immunotherapy target for patients with KIRC.

## Introduction

Kidney neoplasms, namely, renal cell carcinomas (RCCs), are common and life-threatening with approximately 431,288 new cases and 179,368 deaths in 2020 (Global Cancer Observatory) (Global Cancer Observatory, 2020). Clear cell RCC (ccRCC), also known as kidney renal clear cell carcinoma (KIRC), is one of the most common histological subtypes of RCC, accounting for approximately 75% of cases (Hsieh et al., 2017). Studies on genes and pathways include VHL, BAP1, FLCN, FH, MET, SDH, TSC1, TSC2, SETD2, PBRM1, PTEN, PI3K, and mTOR. Owing to the heterogeneity of KIRC, it shows an aggressive phenotype, such as metastasis to distant organs (Linehan and Ricketts, 2019). Recently, Zhang et al. (2019a) indicated that tumor-infiltrating immune cells are important prognostic factors of RCC and immunomodulatory molecules are associated with the poor prognosis in KIRC. Tang et al. (2021) and Tang et al. (2022) reported that ferroptosis biomarkers may predict papillary RCC prognosis and guide treatment options and previously found 14 pyroptosis-related lncRNA-based signatures as robust prognostic and predictive tools for overall survival (OS) in ccRCC. However, there is limited knowledge regarding biomarkers that are correlated with immune infiltration and immunomodulatory molecules as prognosis and therapeutic targets in KIRC. Therefore, there is an urgent need to explore useful biomarkers for the prognosis and immunotherapy of KIRC.

Calreticulin (CALR), an endoplasmic reticulum (ER)-resident Ca^2+^-binding chaperone protein, regulates the protein folding quality control pathways. CALR mechanistically contributes to the initiation of adaptive anticancer immunity in the context of immunogenic cell death, a functional variant of regulated cell death that is sufficient to elicit an antigen-specific immune response in immunocompetent cells (Galluzzi et al., 2017; Ren et al., 2019; Galluzzi et al., 2020). As a reticular protein, a quality control system for the newly synthesized proteins and glycoproteins involves CALR, which relies on multiple additional chaperones, such as protein disulfide isomerase family A member 3 (PDIA3) (Rutkevich and Williams, 2011). In the past decade, accumulated data have indicated that CALR is a major determinant of cellular adjuvanticity, that is, the ability of stressed and dying cells to deliver costimulatory (rather than coinhibitory) signals to immune cells (Galluzzi et al., 2017; Galluzzi et al., 2020). Previous studies have shown that CALR is associated with various diseases, such as myeloproliferative neoplasms (MPNs) (Klampfl et al., 2013; Nangalia et al., 2013), neuroblastoma (Hsu et al., 2005), colorectal carcinoma (Peng et al., 2010), non-small cell lung carcinoma (NSCLC) (Fucikova et al., 2016), acute myeloid leukemia (AML) (Schardt et al., 2009), osteosarcoma (Zhang et al., 2017), and glioblastoma (Muth et al., 2016). Nevertheless, there are no studies on the prognostic or predictive value of CALR in KIRC.

This study systematically evaluated the prognostic value of CALR expression in patients with KIRC using The Cancer Genome Atlas (TCGA) database, Clinical Proteomics Tumor Analysis Consortium (CPTAC), the Human Protein Atlas (HPA), Tumor Immune Estimation Resource (TIMER), and TIMER2.0. Additionally, the STRING database, gene set enrichment analysis (GSEA), gene ontology (GO) analysis, and Kyoto Encyclopedia of Genes and Genomes (KEGG) pathway analysis were used for functional assessment. Lastly, we analyzed the correlation between CALR and immune infiltration levels in tumor microenvironments in KIRC using the TIMER2.0 database with single-sample GSEA (ssGSEA), and confirmed the results using the Gene Expression Profiling Interactive Analysis (GEPIA) database.

## Materials and methods

### Tumor Immune Estimation Resource database analysis

TIMER (https://cistrome.shinyapps.io/timer/) is a website containing comprehensive resources on immune infiltrates across diverse cancer types (Li et al., 2016; Li et al., 2017). TIMER2.0 (http://timer.cistrome.org/) is the latest version of TIMER which provides multiple immune deconvolution methods for estimating the abundance of immune infiltration and allows users to comprehensively explore tumor immunological, clinical, and genomic features (Li et al., 2020). The CALR expression in diverse human cancers was assessed by TIMER crossing TCGA databases. CALR expression levels are displayed in tumors compared with normal tissues for each cancer type using box plots, as shown in gray columns when normal data are available. The Wilcoxon test was used to evaluate the statistical significance of differential expression.

### Data source collection

TCGA (https://portal.gdc.cancer.gov/) is a comprehensive database containing gene expression data and corresponding clinical information. In this study, the data on gene expression and clinical information (workflow type: HTSeq-FPKM, level 3) were identified and downloaded from the TCGA-KIRC project. This project contains 611 files (RNA-seq) with 530 KIRC cases and 72 normal kidneys. A total of four KIRC cases were replicated twice, and one KIRC case was replicated once, and they were excluded from the analysis. Based on previous studies (Dudani et al., 2021; Yu et al., 2021), the age was grouped with a cut-off of 60 years.

### Protein expression level analysis

UALCAN (http://ualcan.path.uab.edu/index.html) (Chandrashekar et al., 2017) is a comprehensive and interactive web resource for analyzing cancer Omics data, and the protein expression for ccRCC is available from the CPTAC dataset (Chen et al., 2019). We analyzed protein expression levels of CALR in KIRC using UALCAN. The analysis was based on sample type and tumor grade. *Z*-values represent standard deviations from the median across samples for the KIRC. Each sample profile from the CPTAC was first normalized by log2 spectral count ratio values and then normalized across samples.

The HPA (https://www.proteinatlas.org/) (Uhlén et al., 2015; Uhlen et al., 2017) database includes a tissue atlas, single-cell type atlas, pathology atlas, brain atlas, blood atlas, and cell atlas. We searched the immunohistochemical images of CALR expressed from the tissue atlas and pathology atlas sections for normal and cancer tissues. We selected samples that were stained with antibody CAB001513; patient ID 1933 and 1943 for normal tissue samples, and two samples from patient ID 679 for renal cancer samples.

### Kaplan–Meier survival analysis

The Kaplan–Meier curve provides survival probability information for patients throughout the study follow-up. The Kaplan–Meier analysis was performed for the OS, disease-specific survival (DSS), and progression-free interval (PFI) of patients with KIRC between the high and low CALR expression groups using the log-rank test. Raw data were obtained from TGCA-KIRC, and a part of the prognostic data from the literature by Liu et al. (2018). Replicated and control samples were excluded from the analysis. Statistical analysis was conducted using the R package survival (version 3.2-10) and graphed using the *R* package survmine (version 0.4.9). Solid lines represent the survival rates and censored data, and dotted lines represent confidence intervals.

### Functional enrichment and analysis

The protein–protein interaction (PPI) network was established using the STRING database (https://string-db.org/) (Szklarczyk et al., 2021). Gene set enrichment analysis (GSEA) was conducted using *R* package clusterProfiler (version 3.14.3) (Yu et al., 2012), and h.all.v7.2. symbols.gmt (Hallmarks) denotes the reference cluster. Based on the GSEA results, 152 genes with absolute log2FC > 2 and padj. < 0.05 were selected for further analysis. GO, including cellular component (CC), biological process (BP), molecular function (MF) (The Gene Ontology), and KEGG (Kanehisa and Goto, 2000) pathway analyses, was also performed using the *R* package clusterProfiler (v3.14.3). The Spearman correlation coefficient test was used for genes correlated with CALR and was performed using the *R* package stat (3.6.3).

### Immune infiltration analysis

The *R* packages GSVA (version 1.34.0) (Hanzelmann et al., 2013) and immune landscape (Bindea et al., 2013) were used to explore the relationship between CALR and immune cells. The TIMER database was used to confirm the correlation between tumor-infiltrating immune cells and CALR in KIRC. In *R* software (version 4.1.0), the CIBERSORT algorithm (Chen et al., 2018) combined with the Homo sapiens gene feature matrix was used to analyze 22 immune cell compositions and their correlation with the expression of CALR in the TCGA-KIRC databases. Furthermore, we evaluated the links between CALR expression and expression patterns of immunomodulators and genes associated with immune cell infiltration (Xiao et al., 2020) using the TIMER2.0 database and *R* package estimate (Yoshihara et al., 2013). GEPIA2 (http://gepia2.cancer-pku.cn/#index) is a web server available for differential gene expression, gene expression profiling, correlation analysis, and survival analysis (Tang et al., 2017). We confirmed the correlation between CALR expression and immunomodulators of GEPIA2.

### Statistical analysis

All statistical analyses were conducted using *R* software (version 3.6.3). RNA-seq data were transformed using log2 (FPKM+1). The Shapiro–Wilk normality test was used to check the data normality. Wilcoxon rank sum test and Wilcoxon signed rank test were used for CALR expression in paired adjacent normal and KIRC tissues. Chi-square and Fisher tests and logistic regression analysis were performed between clinical characteristics and CALR expression. Kruskal–Wallis test and Dunn’s test used for CALR expression in KIRC are stratified by different clinical characteristics. Cox regression for univariate and multivariate analyses showed that clinical characteristics were associated with the OS. The degree of correlation was analyzed using the Spearman’s correlation coefficient. Figures and tables were constructed using the *R* package ggplot2 (version 3.3.3) and xiantao platforms (https://www.xiantao.love/products). *p < 0.05, **p < 0.01, and ***p < 0.001 were considered statistically significant.

## Results

### Expression levels of calreticulin in different types of human cancers

**FIGURE 1:**
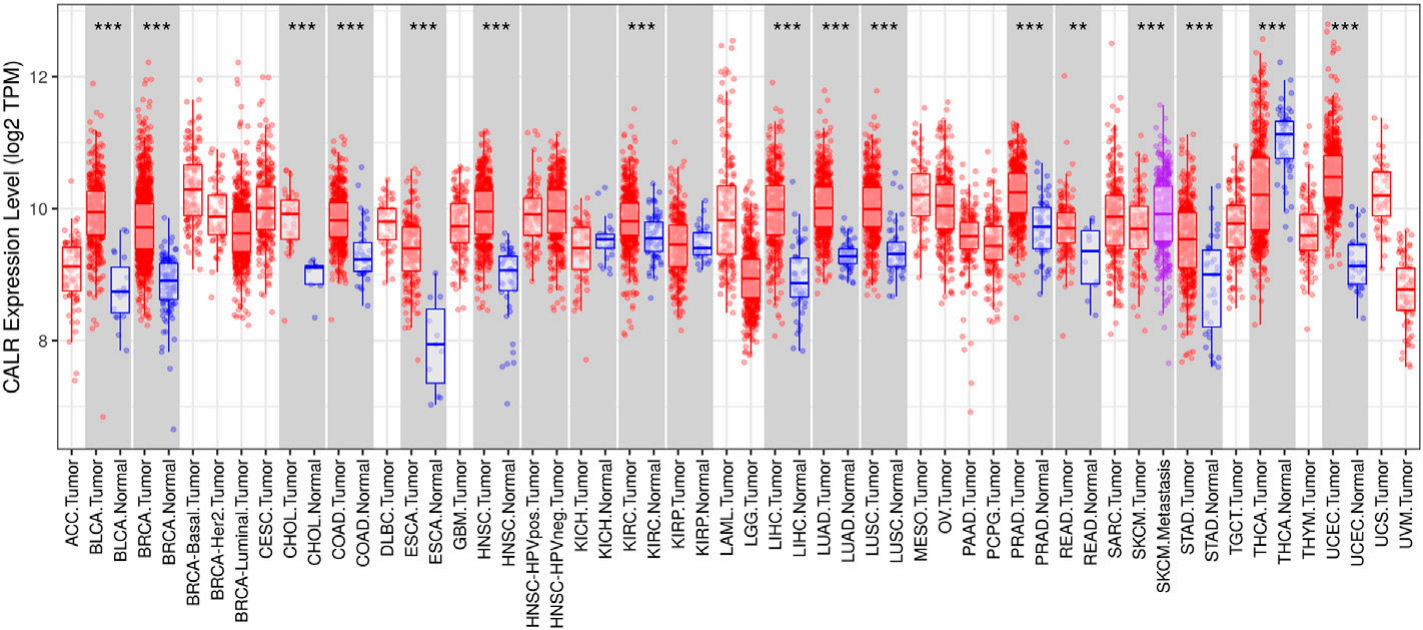
CALR expression in different types of tumor and normal tissues in the TIMER database. (*p < 0.05, **p < 0.01, ***p < 0.001).

To evaluate CALR expression in various human cancers, we examined the CALR expression in multiple cancers using the TIMER database. The differential expression of CALR between the tumor and adjacent normal tissues across all TCGA tumors is shown in Figure 1. The CALR expression was significantly higher in BLCA, BRCA, CHOL, COAD, ESCA, HNSC, KIRC, LIHC, LUAD, LUSC, PRAD, READ, STAD, and UCEC than in adjacent normal tissues. Interestingly, the CALR expression was significantly higher in SKCM metastasis than in SKCM tumor tissues, and was significantly lower in THCA than in adjacent normal tissues.

### High expression level of calreticulin in kidney renal clear cell carcinoma

**FIGURE 2:**
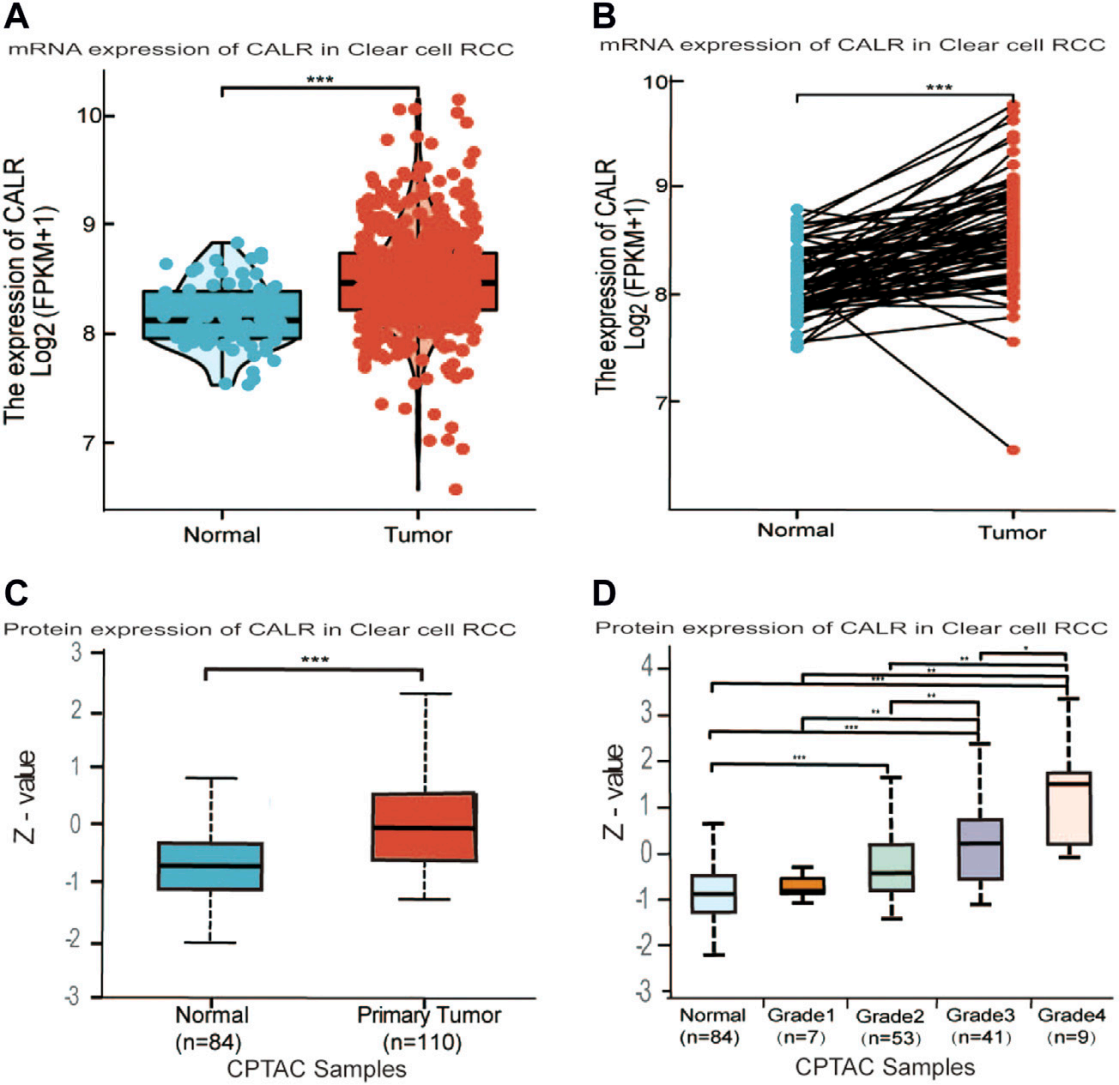
Differential expression analysis of CALR in KIRC. **(A,B)** CALR mRNA expression in normal and tumor tissues. **(C,D)** Protein level expression of CALR in KIRC.

**FIGURE 3:**
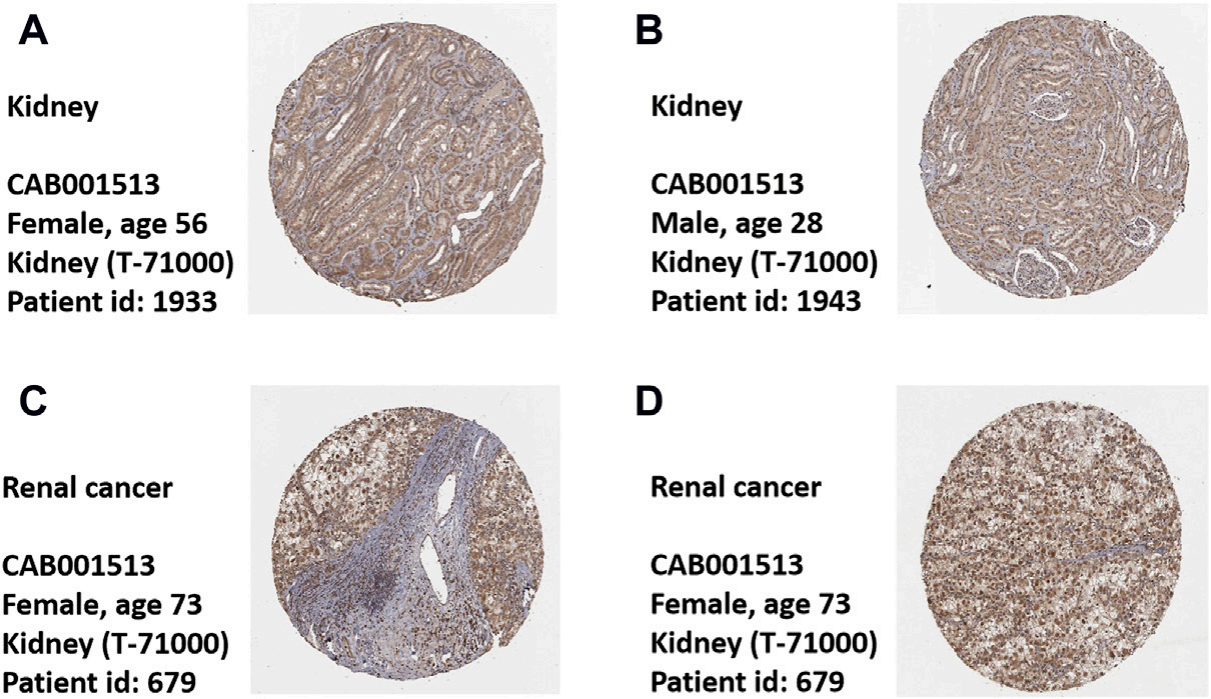
Expression of CALR in healthy control and KIRC patient’s tissues. **(A,B)** Representative images of immunohistochemistry showing CALR expression in normal kidney tissues. **(C,D)** Representative images of immunohistochemistry showing CALR expression in KIRC patient’s tissues.

To comprehensively analyze the expression level of CALR in KIRC, we analyzed its mRNA and protein expression using TCGA, CPTAC, and HPA databases. The TCGA database showed that the expression of CALR mRNA was significantly higher in KIRC tissues than in normal tissues (Figure 2A). Meanwhile, CALR was upregulated in KIRC tumors compared to that in paired adjacent normal tissues (Figure 2B). Additionally, the protein expression of CALR in KIRC from the CPTAC database was significantly increased in tumors compared with those in normal tissues (Figure 2C) and significantly increased with tumor grade (Figure 2D). Protein expression levels were further confirmed using HPA databases. CALR expression was not detected in normal kidney tissues (Figures 3A,B), and was low in cancer tissues (Figures 3C,D).

Generally, the abovementioned results showed that CALR was overexpressed in KIRC in both the mRNA and protein expression levels.

### Association between calreticulin and clinical characteristics in the Cancer Genome Atlas-kidney renal clear cell carcinoma

**FIGURE 4:**
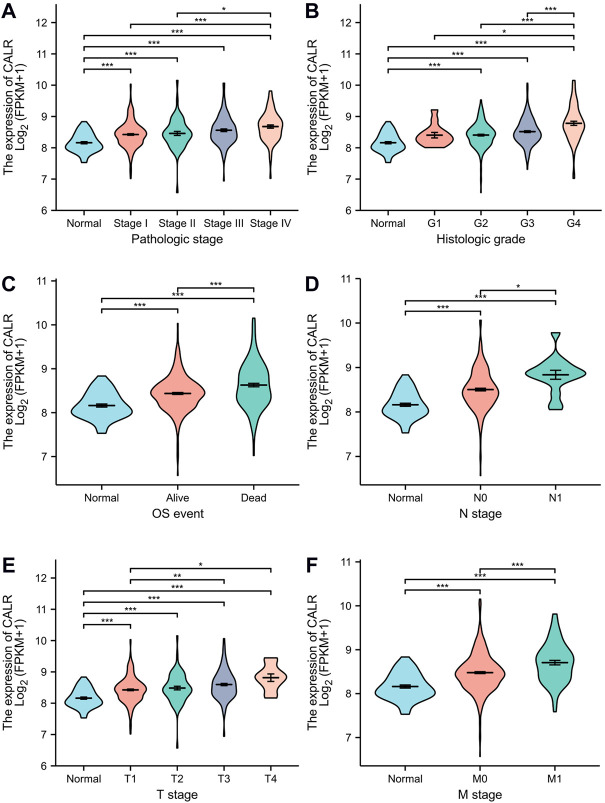
Violin plot evaluating CALR expression of patients with KIRC according to different clinical characteristics. **(A)** Pathologic stage; **(B)** histologic grade; **(C)** OS event; **(D)** N stage; **(E)** T stage; and **(F)** M stage (*p < 0.05, **p < 0.01, ***p < 0.001).

To investigate the association between CALR and clinical characteristics, we first analyzed the clinical characteristics of patients with KIRC classified by CALR (Table 1). The CALR expression level was divided by the median CALR expression. Remarkably, the T stage, N stage, M stage, pathological stage, and histological grade showed significant differences between the high CALR expression groups and low CALR expression groups. To further investigate the clinical significance of CALR expression, we analyzed TCGA-KIRC clinical data. As shown in Figure 4, an increased CALR in KIRC tissues was significantly correlated with consistent clinical characteristics. For example, CALR significantly increased gradually with the pathological stage (Figure 4A) and historical grade (Figure 4B). CALR was also strongly correlated with the overall survival (Figure 4C), N stage (Figure 4D), T stage (Figure 4E), and M stage (Figure 4F).

**TABLE 1:** Relationship between the expression of CALR and clinical characteristics.

Characteristic	Low expression of CALR	High expression of CALR	*p*	Statistical method
*n*	265	265		
Age, *n* (%)			0.543	Chisq.test
≤60	136 (25.7%)	128 (24.2%)		
>60	129 (24.3%)	137 (25.8%)		
Gender, *n* (%)			0.649	Chisq.test
Female	90 (17%)	96 (18.1%)		
Male	175 (33%)	169 (31.9%)		
T stage, *n* (%)			0.004	Chisq.test
T1	152 (28.7%)	119 (22.5%)		
T2	36 (6.8%)	33 (6.2%)		
T3	75 (14.2%)	104 (19.6%)		
T4	2 (0.4%)	9 (1.7%)		
N stage, *n* (%)			0.038	Chisq.test
N0	120 (22.6%)	119 (22.5%)		
N1	3 (0.5%)	13 (2.5%)		
NA/missing	142 (26.8%)	133 (25.1%)		
M stage, *n* (%)			0.001	Chisq.test
M0	219 (41.3%)	201 (37.9%)		
M1	25 (5%)	53 (10%)		
NA/missing	21 (4%)	11 (2.0%)		
Pathologic stage, *n* (%)			0.002	Chisq.test
Stage I	150 (28.3%)	115 (21.7%)		
Stage II	31 (5.9%)	26 (4.9%)		
Stage III	56 (10.6%)	67 (12.6%)		
Stage IV	27 (5.1%)	55 (10.4%)		
NA/missing	1 (0.1%)	2 (0.4%)		
Primary therapy outcome, *n* (%)			0.065	Fisher’s.test
PD	6 (1.1%)	5 (0.9%)		
SD	4 (0.8%)	1 (0.1%)		
PR	0 (0%)	2 (0.4%)		
CR	70 (13.2%)	50 (9.5%)		
NA/missing	185 (34.9%)	207 (39.1%)		
Histologic grade, *n* (%)			<0.001	Fisher’s.test
G1	10 (1.9%)	4 (0.8%)		
G2	129 (24.3%)	98 (18.5%)		
G3	102 (19.2%)	104 (19.6%)		
G4	19 (3.6%)	56 (10.6%)		
NA/missing	5 (0.9%)	3 (0.6%)		
Serum calcium, *n* (%)			0.155	Chisq.test
Elevated	7 (1.3%)	3 (0.6%)		
Low	110 (20.8%)	93 (17.5%)		
Normal	66 (12.5%)	84 (15.8%)		
NA/missing	82 (15.5%)	85 (16.0%)		
Hemoglobin, *n* (%)			0.119	Fisher’s.test
Elevated	4 (0.8%)	1 (0.1%)		
Low	123 (23.2%)	138 (26.0%)		
Normal	90 (17.0%)	94 (17.8%)		
NA/missing	48 (9.1%)	32 (6.0%)		
Laterality, *n* (%)			0.931	Fisher’s.test
Left	124 (23.4%)	125 (23.6%)		
Right	141 (26.7%)	139 (26.2%)		
NA/missing	0 (0%)	1 (0.1%)		

### Calreticulin is upregulated in kidney renal clear cell carcinoma and correlated with poor survival

**FIGURE 5:**
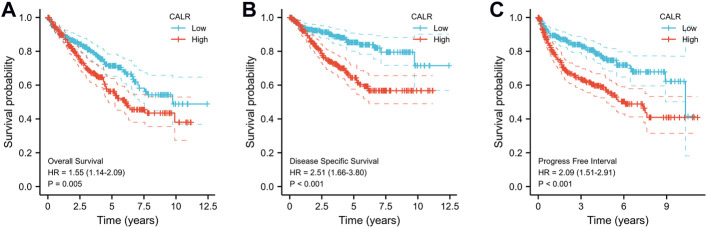
Kaplan–Meier survival curves comparing high and low expression of CALR in KIRC from TCGA databases. **(A)** K–M curves of OS for CALR in KIRC; **(B)** K–M curves of DSS for CALR in KIRC; **(C)** K–M curves of PFI for CALR in KIRC.

The Kaplan–Meier survival analysis of CALR predicted the prognostic value in KIRC patients from TCGA databases, and the association between CALR expression and OS, DSS, and PFI was analyzed. As shown in Figure 5, a high CALR expression was significantly associated with poor OS (*p* = 0.005), DSS (*p* < 0.001), and PFI (*p* < 0.001) in KIRC. Univariate Cox analysis demonstrated that high CALR expression was significantly correlated with poor overall survival [hazard ratio (HR) = 1.541, 95% CI = 1.137–2.087, *p* = 0.005]. However, multivariate Cox analysis showed that CALR expression was associated with OS but not an independent risk factor in patients (HR = 0.899, 95% CI = 0.567–1.424, *p* = 0.65), as shown in Table 2 and Supplementary Figures S1, S2.

**TABLE 2:** Univariate and multivariate Cox regression analyses of clinical characteristics associated with the overall survival.

Characteristic	Total (*N*)	Univariate analysis		Multivariate analysis	
		Hazard ratio (95% CI)	*p*-value	Hazard ratio (95% CI)	*p*-value
Age (>60 vs. ≤60)	530	1.753 (1.290–2.383)	<0.001	1.708 (1.107–2.635)	0.016
Gender (female vs. male)	530	0.951 (0.697–1.296)	0.750		
T stage (T3 and T4 vs. T1 and T2)	530	3.160 (2.332–4.283)	<0.001	1.572 (0.691–3.577)	0.281
N stage (N1 vs. N0)	255	3.426 (1.818–6.456)	<0.001	1.875 (0.922–3.813)	0.082
M stage (M1 vs. M0)	498	4.333 (3.170–5.922)	<0.001	2.808 (1.628–4.843)	<0.001
Pathologic stage (Stage III and IV vs. Stage I and II)	527	3.860 (2.809–5.305)	<0.001	1.237 (0.479–3.192)	0.661
Histologic grade (G3 and G4 vs. G1 and G2)	522	2.660 (1.888–3.748)	<0.001	1.722 (1.043–2.840)	0.033
Laterality (right vs. left)	529	0.706 (0.523–0.952)	0.023	1.133 (0.730–1.756)	0.614
CALR (high vs. low)	530	1.546 (1.141–2.095)	0.005	0.924 (0.595–1.434)	0.724

Thus, CALR expression is associated with the clinical characteristics of patients with KIRC. A high CALR expression was correlated with advanced disease stages and grades and poor OS, DSS, and PFI.

### Functional and enrichment analysis of calreticulin

**FIGURE 6:**
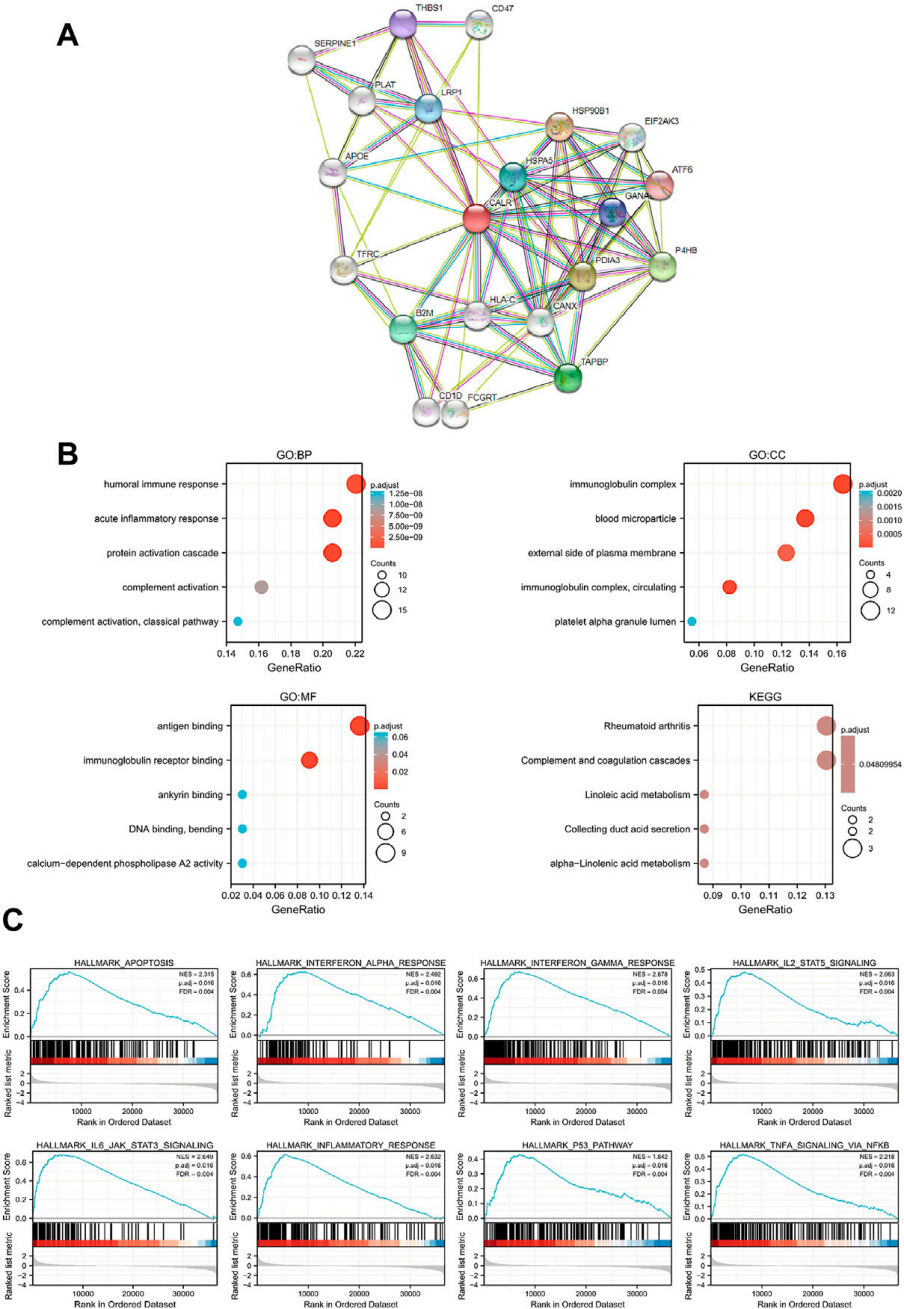
Functional and enrichment analysis of CALR in TCGA-KIRC. **(A)** PPI network of CALR and related proteins. **(B)** GO and KEGG analysis of CALR in KIRC. **(C)** Enrichment plots from GSEA.

To predict the function of CALR and associated protein interaction network, we performed GSEA, GO, and KEGG pathway analyses using TCGA-KIRC data. The PPI network was downloaded from the STRING database (Figure 6A). GO enrichment analysis indicated that crosstalk with the humoral immune response and acute inflammatory response BPs, cellular components refer to immunoglobulin complex and blood microparticles, and MFs mainly represent antigen binding and immunoglobulin receptor binding. The KEGG pathway analysis showed that the main enrichment pathways were rheumatoid arthritis and complement and coagulation cascades (Figure 6B). For GSEA, FDR (*q*-value) < 0.25, p. adjust <0.05 was considered significant enrichment, and CALR was enriched in 37 gene sets (Supplementary Table S1). As shown in Figure 6C, CALR was enriched in IL-6 and IL-2 signaling, interferon signaling, TNF signaling, inflammatory response, apoptosis, and the p53 pathway. Moreover, the Spearman correlation coefficient test showed that 30,043 genes were coexpressed with CALR in TGCA-KIRC data, and the top 50 genes that are most positively and negatively associated with CALR are shown in a heatmap (Supplementary Figure S3). We found 37 pathways with significant differences between low expression (group A) and high expression (group B) of CALR in TGCA-KIRC databases, as shown in a heatmap and grouped comparison graph (Supplementary Figure S5).

### Calreticulin expression is correlated with immune infiltration level in kidney renal clear cell carcinoma

**FIGURE 7:**
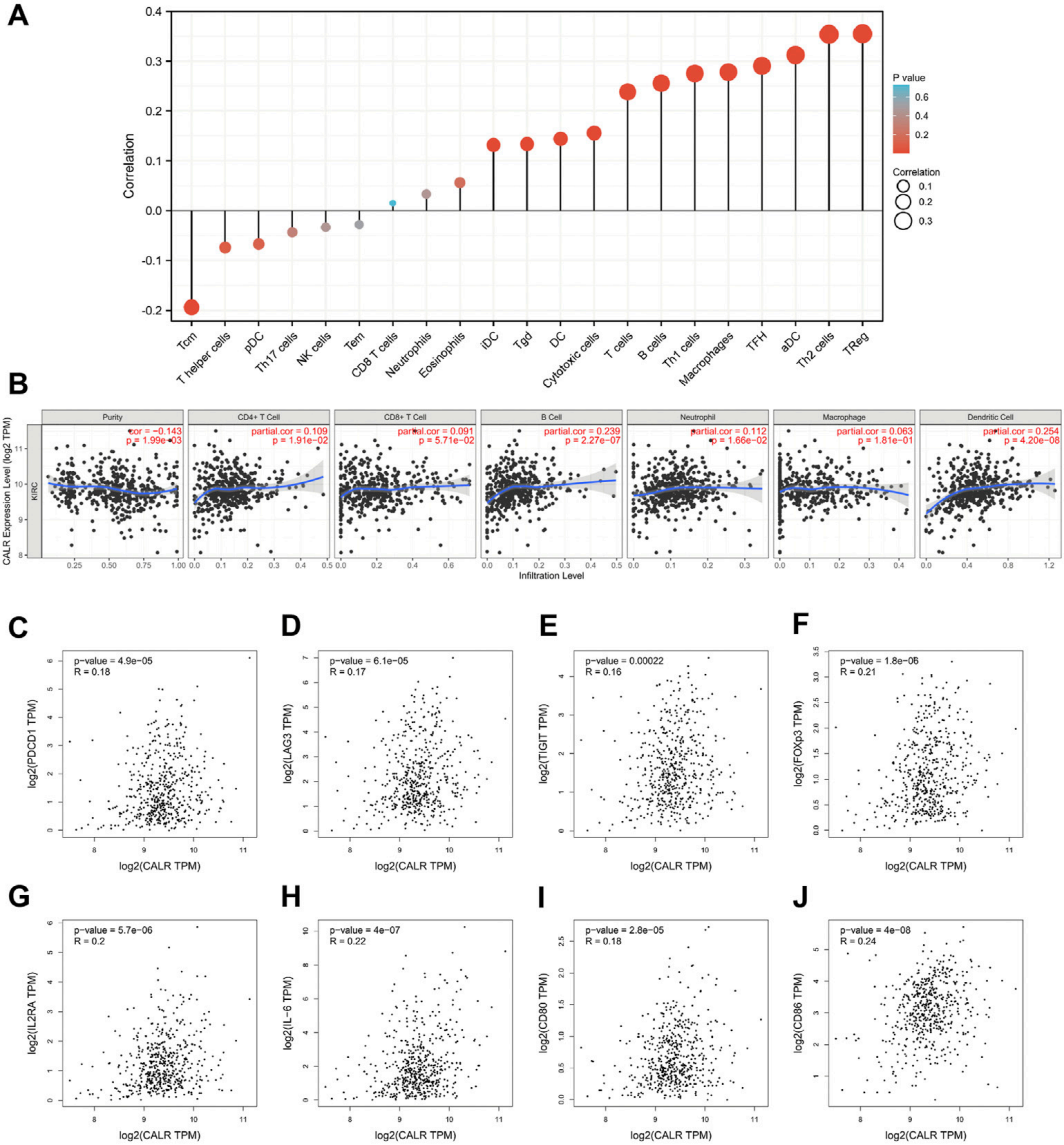
Correlation between CALR expression and immune infiltration in TCGA-KIRC. **(A)** Correlation between CALR expression and immune cells. **(B)** Correlation between CALR expression and immune cells after purity adjustment. **(C–J)** Correlation between CALR expression and immunomodulatory molecules.

To explore the association between CALR and immune cells and molecules, we further assessed the immune cell infiltration of TCGA-KIRC and found that Treg, Th2 cells, aDC, TFH, macrophages, Th1 cells, B cells, T cells, cytotoxic cells, DC, Tgd, and iDC infiltration levels were positively correlated with the CALR expression, whereas Tcm was negatively correlated with the CALR expression (Figure 7A; Supplementary Table S2). We validated that the expression of CALR was correlated with 21 immune cells, and the infiltrating abundance of Tregs was most positively correlated with the CALR expression, while the resting infiltrating abundance of mast cells was most negatively correlated with the CALR expression in TGCA-KIRC databases (Supplementary Figure S6A). ESTIMATEScore, ImmuneScore, StromalScore, and TumorPurity were calculated and are shown in Supplementary Figures S6B–E. Additionally, we confirmed the CALR expression after purity adjustment using TIMER (Figure 7B) and found that CD4^+^ T cells, B cells, neutrophils, and dendritic cells were significantly correlated with CALR in KIRC. Furthermore, we explored the relationship between CALR expression, immunomodulators, and genes associated with immune cell infiltration using TIMER2.0. The results were adjusted for tumor purity, revealing a significant correlation between CALR expression and immunomodulators (PD-1, LAG3, TIGIT, IL-2R, IL-6, CD80, and CD28), Treg markers (FOXP3, CCR8, and TGFβ), Th2 markers (STAT5A and IL-13), TFH markers (BCL6 and IL-21), Th1 markers (STAT1), T cell markers (CD3D, CD3E, and CD2), CD8+T cell markers (CD8A and CD8B), Th17 markers (STAT3), macrophage markers (COX2, CD163, and VSIG4), neutrophil markers (CD11b, CD66b, and CCR7), NK cell markers (KIR2DL4), and DC markers (HLA-DPB1, HLA-DRA, HLA-DPA1, BCDA‐A, and BDCA‐4) in KIRC (Table 3). CALR expression was significantly correlated with immunomodulators in KIRC. Therefore, we further confirmed the correlation between CALR and immunomodulators, such as PD-1, LAG3, TIGIT, and IL-6, using GEPIA2 (Figures 7C–J).

**TABLE 3:** Correlation analysis between CALR and related genes and markers of immune cells in TIMER2.0.

Description	Gene marker	KIRC			
		None		Purity	
		rho	*p*	rho	*p*
Immunomodulators	CTLA4	0.078	0.072	0.067	0.148
	PD‐1(PDCD1)	0.177	***	0.165	***
	BTLA	0.135	**	0.069	0.138
	LAG3	0.185	***	0.166	***
	TIM‐3(HAVCR2)	0.052	0.228	−0.020	0.670
	TIGIT	0.185	***	0.145	**
	IL-2RA	0.193	***	0.136	**
	IL-6	0.214	***	0.212	***
	CD80	0.177	***	0.149	**
	ICOS	0.117	**	0.073	0.120
	CD28	0.195	***	0.147	**
	GZMB	0.079	0.069	0.062	0.186
Treg	FOXP3	0.224	***	0.210	***
	CCR8	0.146	***	0.104	*
	STAT5B	−0.006	0.891	−0.063	0.178
	TGFβ(TGFB1)	0.313	***	0.260	***
Th2	GATA3	0.028	0.518	0.044	0.347
	STAT6	0.011	0.802	−0.016	0.729
	STAT5A	0.173	***	0.105	*
	IL13	−0.125	**	−0.112	*
Tfh	BCL6	0.160	***	0.185	***
	IL21	0.133	**	0.118	*
Th1	T‐bet (TBX21)	0.010	0.811	−0.037	0.426
	STAT4	0.026	0.545	−0.006	0.905
	STAT1	0.259	***	0.205	***
	IFN‐γ(IFNG)	0.124	**	0.089	0.056
	TNF‐α(TNF)	0.046	0.294	0.011	0.811
T cell	CD3D	0.165	***	0.123	**
	CD3E	0.183	***	0.142	**
	CD2	0.150	***	0.105	*
B cell	CD19	0.104	*	0.065	0.165
	CD79A	0.127	**	0.079	0.088
CD8+T cell	CD8A	0.168	***	0.127	**
	CD8B	0.167	***	0.127	**
Th17	STAT3	0.265	***	0.207	***
	IL17A	−0.031	0.479	−0.029	0.540
Macrophages	INOS(NOS2)	0.025	0.567	−0.047	0.310
	IRF5	0.072	0.095	0.048	0.308
	COX2(PTGS2)	0.108	*	0.092	*
	CD163	0.200	***	0.118	*
	VSIG4	0.308	***	0.239	***
	MS4A4A	0.162	***	0.084	0.071
Neutrophils	CD66b (CEACAM8)	−0.142	**	−0.146	**
	CD11b (ITGAM)	0.247	***	0.194	***
	CCR7	0.179	***	0.131	**
Natural killer cells	KIR2DL1	0.015	0.721	−0.014	0.761
	KIR2DL3	0.046	0.291	0.027	0.569
	KIR2DL4	0.119	**	0.112	*
	KIR3DL1	−0.004	0.920	−0.021	0.650
	KIR3DL2	0.058	0.181	0.073	0.117
	KIR3DL3	0.066	0.128	0.066	0.160
	KIR2DS4	0.054	0.214	0.047	0.310
Dendritic cells	HLA‐DPB1	0.225	***	0.160	**
	HLA‐DQB1	0.132	**	0.090	0.053
	HLA‐DRA	0.206	***	0.137	**
	HLA‐DPA1	0.209	***	0.145	**
	BCDA‐1(CD1C)	−0.016	0.708	−0.111	*
	BDCA‐4(NRP1)	0.191	***	0.114	*
	CD11c (ITGAX)	0.023	0.594	0.002	0.973

rho: *R* value of Spearman’s correlation; None: correlation without adjustment; Purity, correlation adjusted by purity. *: *p* < 0.05, **: *p* < 0.01, ***: *p* < 0.001.

Briefly, these results indicated that CALR expression was enriched in immune regulation in KIRC and correlated with immune cell subsets and immunomodulatory markers within tumors. Thus, these results highlight the ability of CALR to regulate immune cell recruitment and activation in KIRC.

## Discussion

In recent studies, CALR has attracted extensive attention owing to its influence on tumor progression, malignant transformation, and response to therapy (Fucikova et al., 2021). For example, CALR is the most frequent mutation in MPNs (Klampfl et al., 2013; Nangalia et al., 2013). Moreover, CALR has been investigated in lung cancer (Fucikova et al., 2016) and pancreatic cancer (Sheng et al., 2020). However, it remains unclear whether abnormal CALR expression influences the KIRC prognosis and immune infiltration. In this study, we investigated the expression of CALR in KIRC *via* bioinformatics analysis, revealed a comprehensive association between CALR and clinical characteristics and outcomes of patients with KIRC, and verified that CALR expression was closely related to immune infiltration in KIRC.

In the present study, we first identified that the CALR mRNA expression level was significantly upregulated in KIRC in the TCGA database and further confirmed the expression of CALR at the protein level. Furthermore, we conducted logistic regression and Kaplan–Meier analysis, which revealed that CALR expression was closely correlated with the TNM stage, pathological stage, historical grade, OS, DSS, and PFI. Kaplan–Meier curves have become a popular analysis tool for survival times (times-to-event) since 1958. In most cases, Kaplan–Meier curves were used to estimate the probability of survival within a given time interval. Defined events or endpoints can express different types of data. Kaplan–Meier curves also make a direct sense of the probability of survival in different groups. However, many factors, such as age, sex, disease stage, and complications, can affect the prognosis and diagnosis of the disease (Rich et al., 2010). In our study, we defined three events or endpoints to explore the prognostic and diagnostic value of CALR in TCGA-KIRC databases. Meanwhile, univariate and multivariate analyses showed that the CALR expression level was a risk factor but not an independent risk factor for OS in patients with KIRC. Previous studies have shown that a high expression of CALR has been linked with improved outcomes in some carcinomas, such as colorectal carcinoma (Peng et al., 2010), NSCLC (Fucikova et al., 2016), AML (Schardt et al., 2009), osteosarcoma (Zhang et al., 2017), glioblastoma (Muth et al., 2016), and ovarian carcinoma (Kasikova et al., 2019). Paradoxically, the robust CALR expression has also been associated with negative prognostic value in some diseases. In particular, a high CALR expression has been correlated with poor outcomes in gastric carcinoma (Chen et al., 2009), NSCLC (Liu et al., 2012), breast carcinoma (Lwin et al., 2010), pancreatic cancer (Matsukuma et al., 2016), neuroblastoma, bladder carcinoma, and mantle cell lymphoma (Chao et al., 2010). Our results are in line with those of previous studies showing that the CALR expression is negatively correlated with the outcome in KIRC. It is important to note that the present results rely on the TGCA-KIRC database. Further research on CALR expression in KIRC should be conducted using larger clinical cohorts.

Second, functional analysis revealed that CALR was enriched in the immune and inflammatory response pathways. CALR closely interacted with PDIA3 (also known as ERp57) and prolyl 4-hydroxylase *β* polypeptide (P4HB, also known as PDIA1), which have been reported as prognostic markers in cervical cancer (Chung et al., 2013), hepatocellular carcinoma (Takata et al., 2016), glioma (Sun et al., 2013), and KIRC (Zhu et al., 2019). In contrast, Zhang et al. (2018) and Zhang et al. (2019b) reported that P4HB was associated with hypoxia, and Ding et al. (2020)found that P4HB mediated inflammation. These results may explain why CALR was enriched in hypoxia and inflammatory pathways in KIRC. These results provided new insights into the mechanisms of KIRC.

Finally, we assessed the correlation between CALR expression and immune cell infiltration. Many studies have demonstrated that immune cells and immunosuppressive checkpoints are significantly related to the poor prognosis in KIRC and that CALR plays an important role in the maintenance of immune cell homeostasis and the regulation of signaling transduction (Hu et al., 2020; Xiong et al., 2020; Fucikova et al., 2021). Huang et al. (2021) and Deng et al. (2021) reported that Tregs and resting mast cells are associated with the disease prognosis and long-term survival in KIRC. Consistent with their results, we found that CALR expression was positively correlated with infiltrating abundance of Tregs, while it was negatively correlated with the resting infiltrating abundance of mast cells in TGCA-KIRC databases. Our results demonstrate that CALR expression is closely related to T cells, subtypes, and dendritic cells. These findings are in accordance with those reported by Geissler et al. (2015) and Qi et al. (2020). Moreover, we found that CALR expression was associated with immunomodulatory markers, such as PD-1 and LAG3. Overall, this result is consistent with the findings of previous studies (Şenbabaoğlu et al., 2016; Hu et al., 2020; Klümper et al., 2020). These results will further help us develop a potential biomarker for the prognosis of KIRC and provide a novel target for immunotherapy for KIRC.

The main limitation of the present study was the lack of real-world studies to support our findings. This limitation is apparent in many similar studies and provides an opportunity for further research. Another limitation of this study is that bioinformatics analysis requires some knowledge of the computational algorithms.

## Conclusion

Our analysis indicated that a high CALR expression has a significantly negative correlation with the disease stage and OS of patients with KIRC. Additionally, our data suggest that CALR is correlated with immune infiltration in KIRC, providing a potential mechanism for immunotherapy. In summary, CALR has been identified as an important biomarker for the KIRC prognostic value and a potential target for immunotherapy.

## Data Availability

Publicly available datasets were analyzed in this study. These data can be found here: https://portal.gdc.cancer.gov/.
