# Supplementary material for: Calreticulin as a prognostic biomarker and correlated with immune infiltrate in kidney renal clear cell carcinoma
Source: Front Genet. 2022 Oct 21;13:909556. doi: 10.3389/fgene.2022.909556 (PMC9633671; doi:10.3389/fgene.2022.909556)
Supplement: Supplementary file 1 [file DataSheet1.PDF]

Supplementary Materials

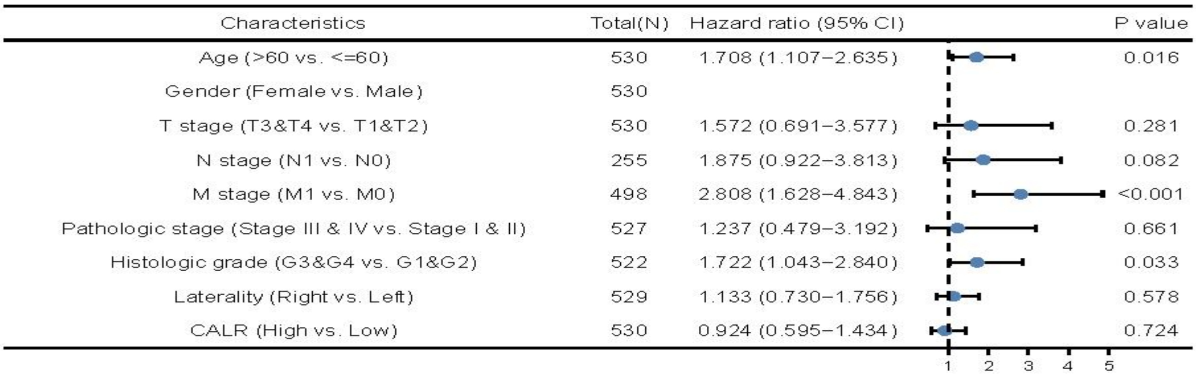

Figure S1: Forest plot of the multivariate Cox regression analysis in KIRC.

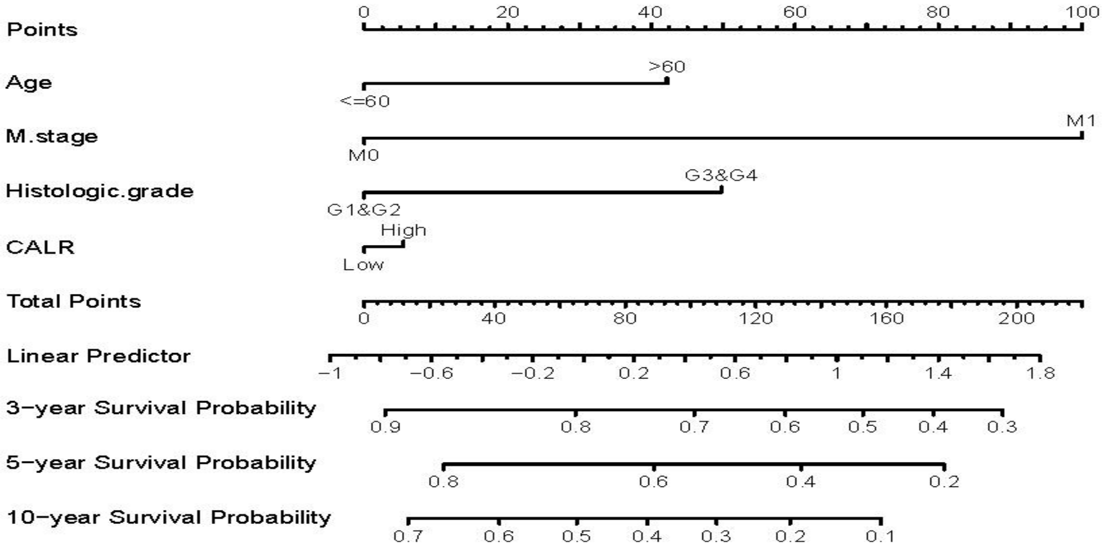

Figure S2 : Nomogram for predicting the probability of patients with 1-, 3- and 5-year overall survival.

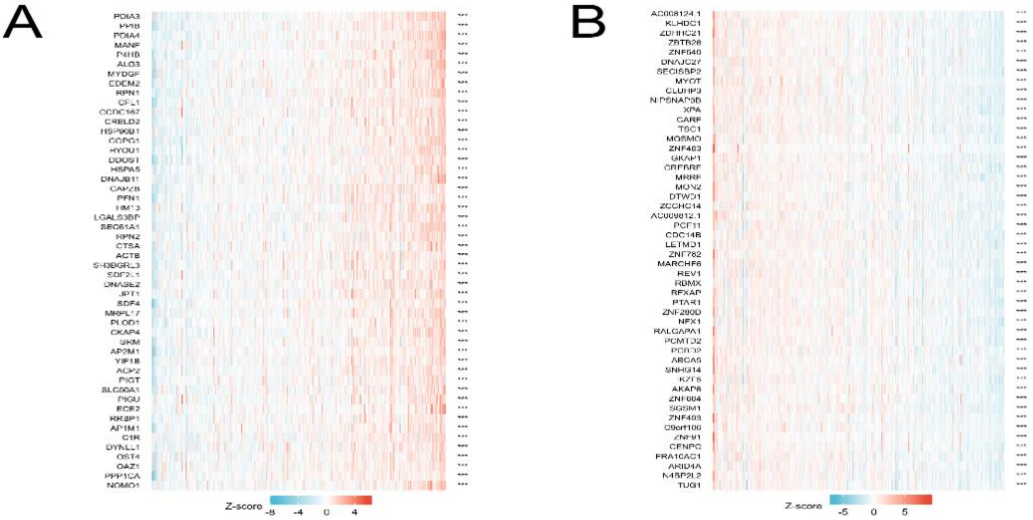

Figure S3 : Genes associated with CALR are shown in a heatmap. (A) Top 50 genes most positively with CALR are shown. (B) Top 50 genes most negatively associated with CALR are shown. *** : p<0.001

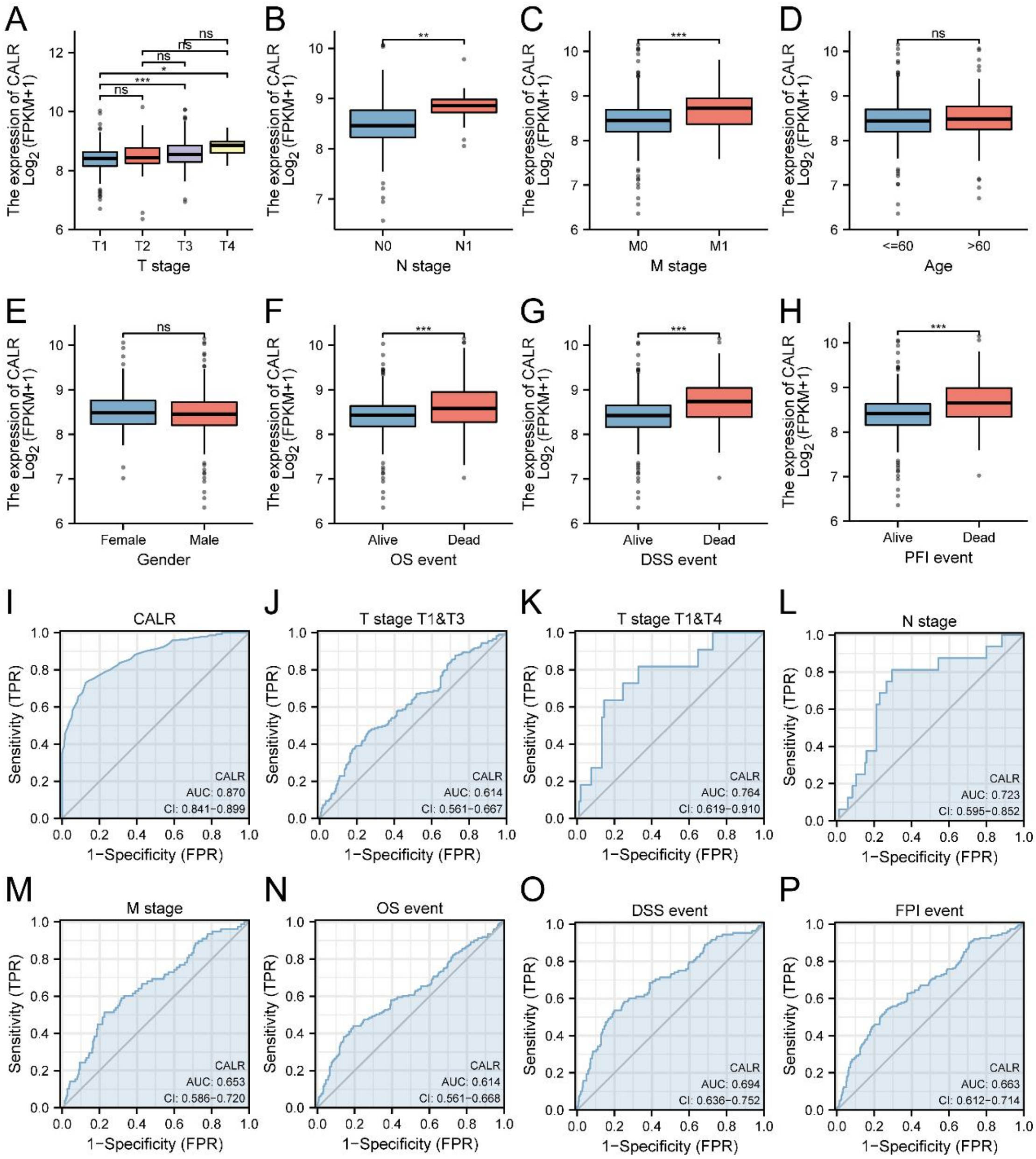

Figure S4 : Expression of CALR in subgroup TGCA-KIRC databases and ROC analysis . (A-H) Different expression of CALR compared in subgroups. (I) ROC curve of CALR in TGCA-KIRC. (J-P) ROC curve of CALR in subgroup in TGCA-KIRC databases. ns: $p \geq 0.05$; *: $p < 0.05$; **: $p < 0.01$; *** : $p<0.001$. AUC: Area Under Curve. OS: Overall Survival. DSS: Disease Specific Survival. PFI: progression-free interval

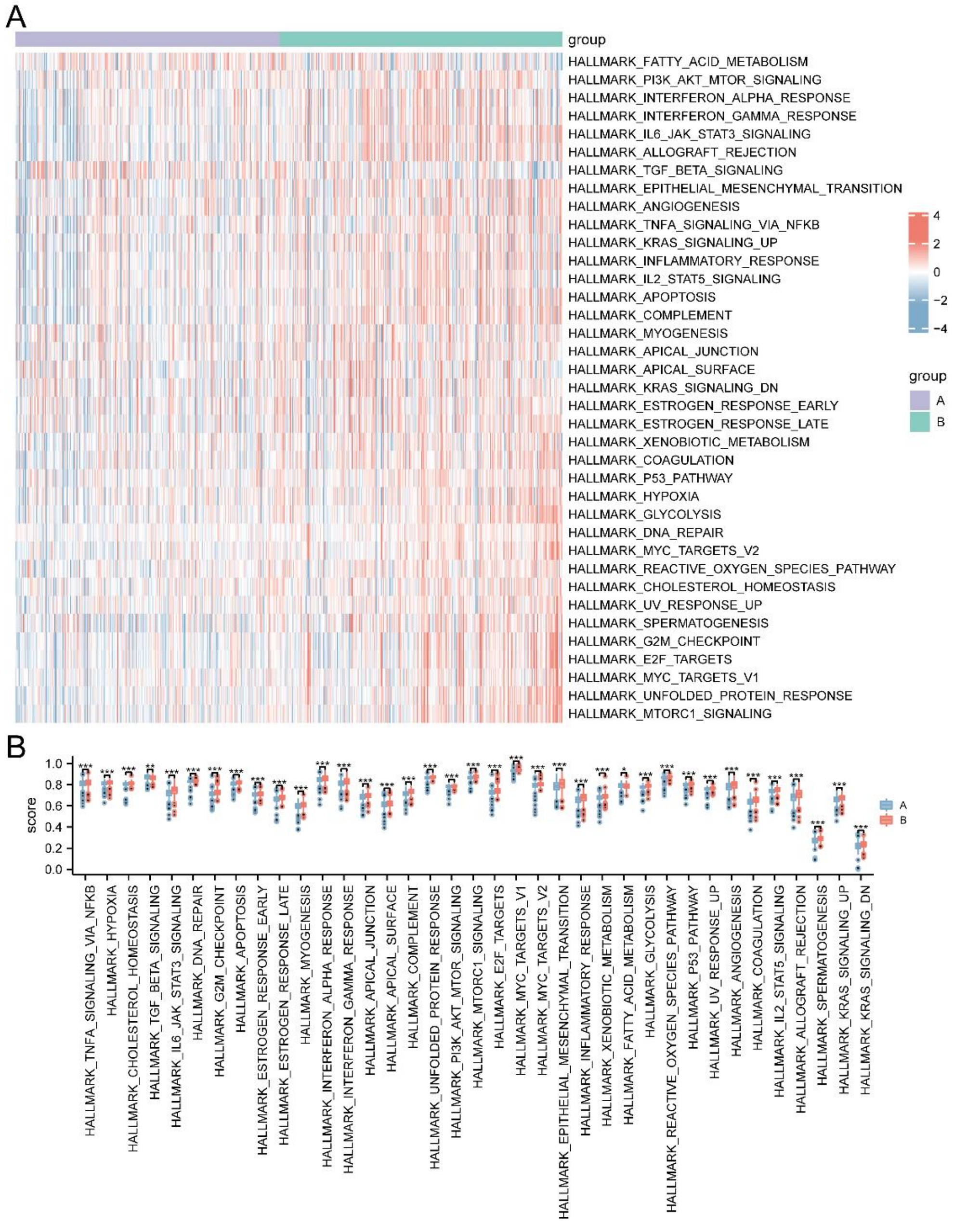

Figure S5 : GSVA enrichment analysis for low and high expression of CALR in TGCA-KIRC databases. (A) A heatmap of CALR in TGCA-KIRC by GSVA. (B) Low and high expression of CALR were compared in 37 pathways. ns: $p \geq 0.05$; *: $p < 0.05$; **: $p < 0.01$; *** : $p<0.001$. group A: low expression of CALR; group B: high expression of CALR.

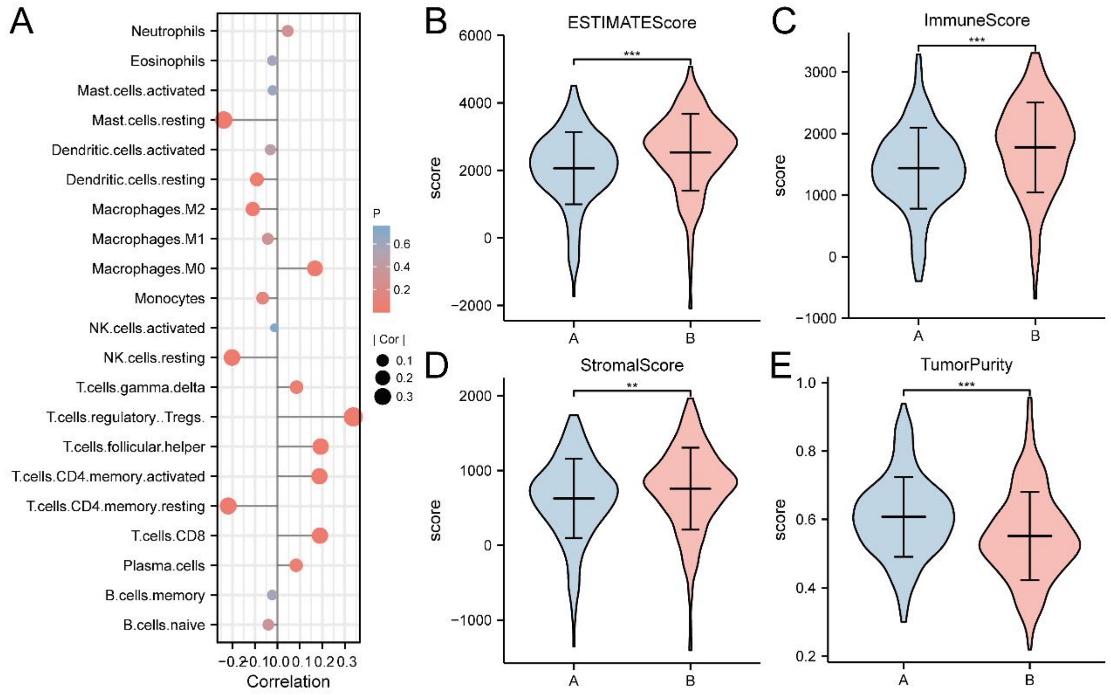

Figure S6 : Correlation between immune infiltration and expression of CALR in TGCA-KIRC databases and immune-related scores. (A) Correlation between immune cell infiltration abundance and CALR in TGCA-KIRC. (B-E) Low and high expression of CALR were compared in ESTIMATEScore, ImmuneScore, StromalScore, and TumorPurity respectively. **: p < 0.01; *** : p<0.001. A: low expression of CALR; B: high expression of CALR.

Table S1: CALR enriched in Gene sets in TGCA-KIRC

Gene Set Name	Size	ES	NES	p.adjust	FDR (qvalue)
HALLMARK_ANGIOGENESIS	36	0.606	2.004	0.016	0.004
HALLMARK_REACTIVE_OXYGEN_SPECIES_PATHWAY	49	0.558	1.947	0.016	0.004
HALLMARK_MYC_TARGETS_V2	58	0.584	2.125	0.016	0.004
HALLMARK_IL6_JAK_STAT3_SIGNALING	87	0.685	2.649	0.016	0.004
HALLMARK_INTERFERON_ALPHA_RESPONSE	97	0.626	2.492	0.016	0.004
HALLMARK_BILE_ACID_METABOLISM	112	0.404	1.643	0.016	0.004
HALLMARK_PEROXISOME	104	0.463	1.851	0.016	0.004
HALLMARK_UNFOLDED_PROTEIN_RESPONSE	111	0.546	2.210	0.016	0.004
HALLMARK_COAGULATION	138	0.662	2.722	0.016	0.004
HALLMARK_DNA_REPAIR	149	0.462	1.911	0.016	0.004
HALLMARK_UV_RESPONSE_UP	158	0.460	1.915	0.016	0.004
HALLMARK_APOPTOSIS	161	0.557	2.315	0.016	0.004
HALLMARK_ALLOGRAFT_REJECTION	200	0.717	3.080	0.016	0.004
HALLMARK_COMPLEMENT	200	0.660	2.834	0.016	0.004

HALLMARK_E2F_TARGETS	200	0.614	2.634	0.016	0.004
HALLMARK_EPITHELIAL_MESENCHYMAL_TRANSITION	200	0.735	3.156	0.016	0.004
HALLMARK_G2M_CHECKPOINT	200	0.586	2.516	0.016	0.004
HALLMARK_INTERFERON_GAMMA_RESPONSE	200	0.670	2.878	0.016	0.004
HALLMARK_KRAS_SIGNALING_UP	200	0.529	2.273	0.016	0.004
HALLMARK_MYC_TARGETS_V1	200	0.519	2.228	0.016	0.004
HALLMARK_TNFA_SIGNALING_VIA_NFKB	200	0.517	2.218	0.016	0.004
HALLMARK_XENOBIOTIC_METABOLISM	200	0.515	2.210	0.016	0.004
HALLMARK_IL2_STAT5_SIGNALING	198	0.483	2.063	0.016	0.004
HALLMARK_MTORC1_SIGNALING	198	0.570	2.436	0.016	0.004
HALLMARK_APICAL_JUNCTION	199	0.519	2.218	0.016	0.004
HALLMARK_ESTROGEN_RESPONSE_EARLY	199	0.361	1.540	0.016	0.004
HALLMARK_ESTROGEN_RESPONSE_LATE	199	0.519	2.216	0.016	0.004
HALLMARK_GLYCOLYSIS	199	0.575	2.456	0.016	0.004
HALLMARK_HYPOXIA	199	0.533	2.274	0.016	0.004
HALLMARK_INFLAMMATORY_RESPONSE	199	0.616	2.632	0.016	0.004
HALLMARK_KRAS_SIGNALING_DN	199	0.324	1.385	0.016	0.004
HALLMARK_MITOTIC_SPINDLE	199	0.354	1.510	0.016	0.004
HALLMARK_MYOGENESIS	199	0.389	1.661	0.016	0.004
HALLMARK_P53_PATHWAY	199	0.431	1.842	0.016	0.004
HALLMARK_PI3K_AKT_MTOR_SIGNALING	104	0.374	1.496	0.019	0.005
HALLMARK_CHOLESTEROL_HOMEOSTASIS	74	0.421	1.584	0.021	0.006
HALLMARK_SPERMATOGENESIS	134	0.337	1.389	0.041	0.011

Abbreviations: ES, enrichment score; NES, normalized enrichment score; FDR, false discovery rate. Gene sets with P.adjust <0.05 and FDR (q-value) <0.25 were considered as significantly enriched.

Table S2: Correlation analysis between CALR and immune cells in TGCA-KIRC

Cell type		Cor.	P
CALR	Treg	0.355	<0.001
	Th2 cells	0.354	<0.001
	aDC	0.313	<0.001
	NK CD56bright cells	0.298	<0.001
	TFH	0.291	<0.001
	Macrophages	0.278	<0.001
	Th1 cells	0.275	<0.001
	B cells	0.256	<0.001
	T cells	0.238	<0.001
	Cytotoxic cells	0.156	<0.001
	NK CD56dim cells	0.147	<0.001
	DC	0.144	<0.001
	Tgd	0.134	0.002
	iDC	0.132	0.002

Eosinophils	0.056	0.193
Neutrophils	0.033	0.441
CD8 T cells	0.015	0.7270
Tem	-0.028	0.516
NK cells	-0.033	0.439
Th17 cells	-0.043	0.316
Mast cells	-0.061	0.155
pDC	-0.067	0.12
T helper cells	-0.074	0.087
Tcm	-0.194	<0.001
