# Supplementary figures and images for: Calreticulin as a prognostic biomarker and correlated with immune infiltrate in kidney renal clear cell carcinoma
Source: Front Genet. 2022 Oct 21;13:909556. doi: 10.3389/fgene.2022.909556 (PMC9633671; doi:10.3389/fgene.2022.909556)

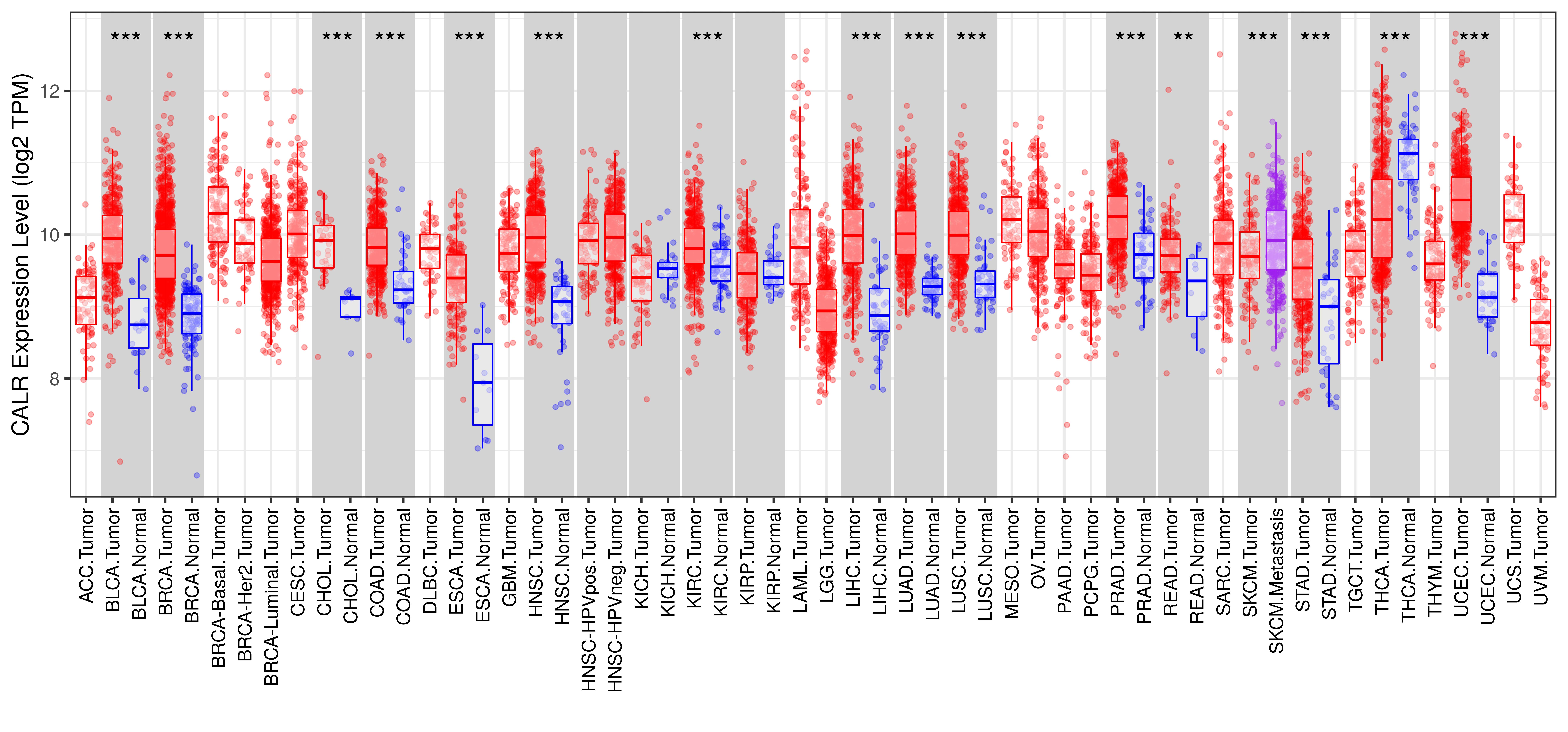

Supplement: Supplementary file 2 [file DataSheet2.ZIP › Figures/Figure 1: Expression analysis of CALR by TIMER databases..jpg]

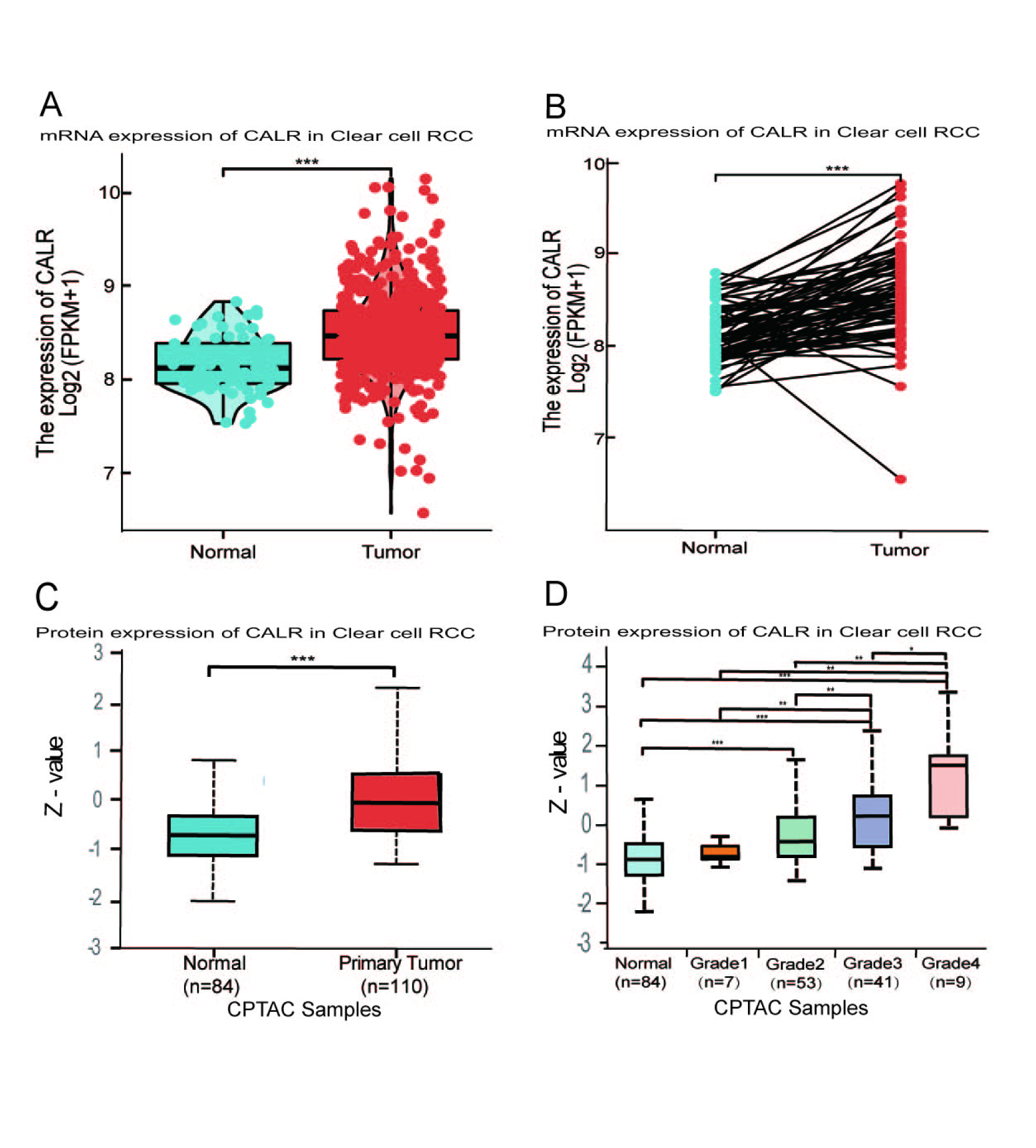

Supplement: Supplementary file 2 [file DataSheet2.ZIP › Figures/Figure 2:Differential expression analysis of CALR in KIRC. (A, B) CALR mRNA expression in normal and tumor tissues. (C, D) Protein level expression of CALR in KIRC..jpg]

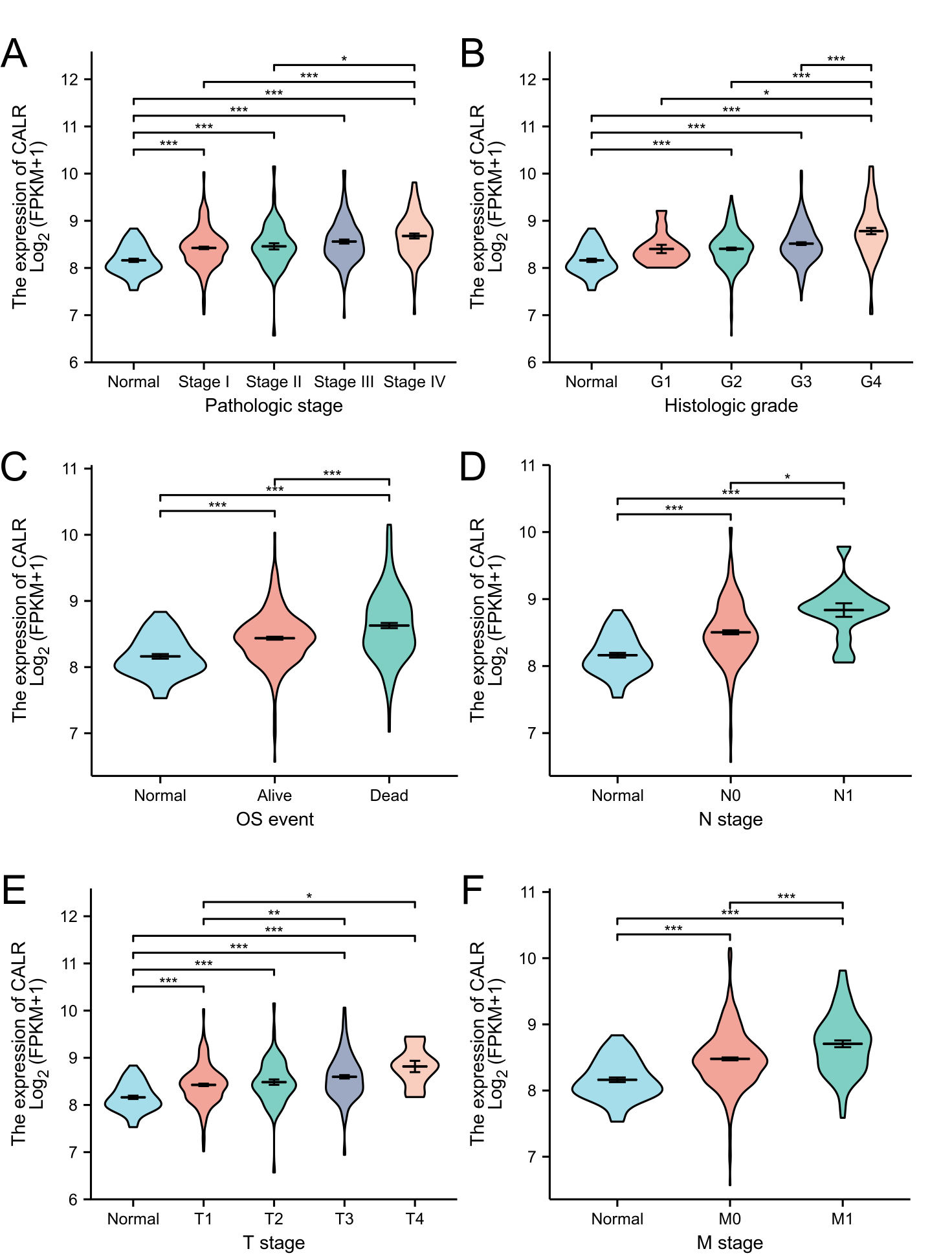

Supplement: Supplementary file 2 [file DataSheet2.ZIP › Figures/Figure 4:violin plot evaluating CALR expression of patients with KIRC according to different clinical characteristics..jpg]

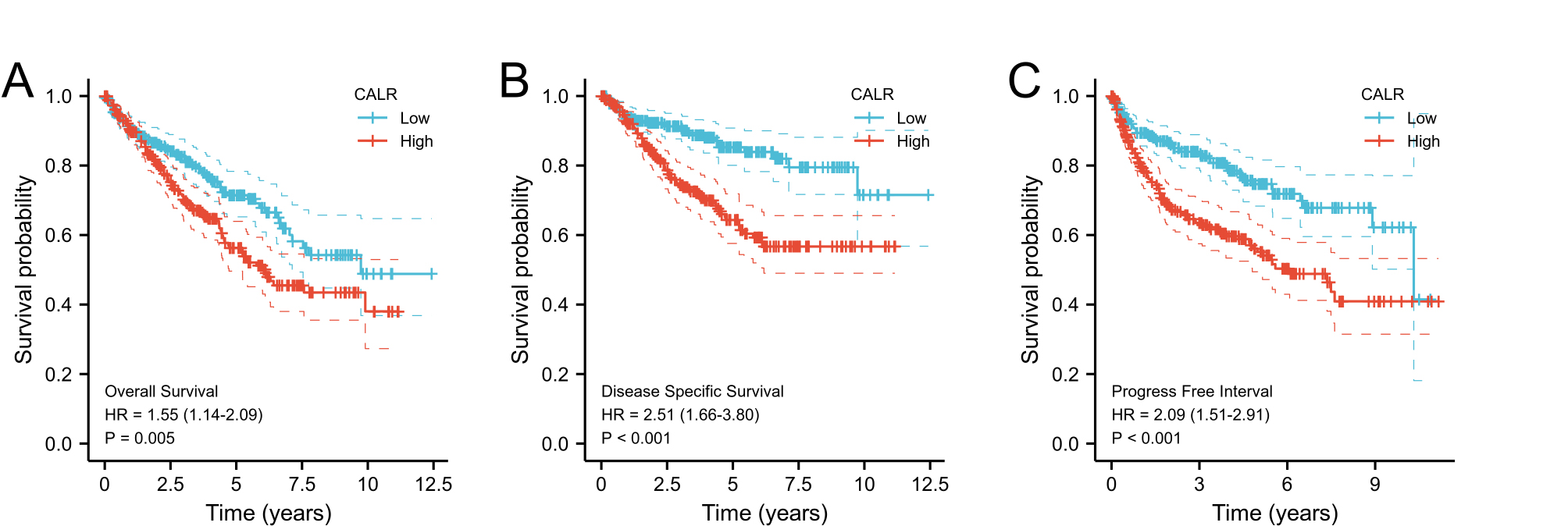

Supplement: Supplementary file 2 [file DataSheet2.ZIP › Figures/Figure 5:Kaplan‐Meier survival curves comparing high and low expression of CALR in KIRC from TCGA databases..jpg]

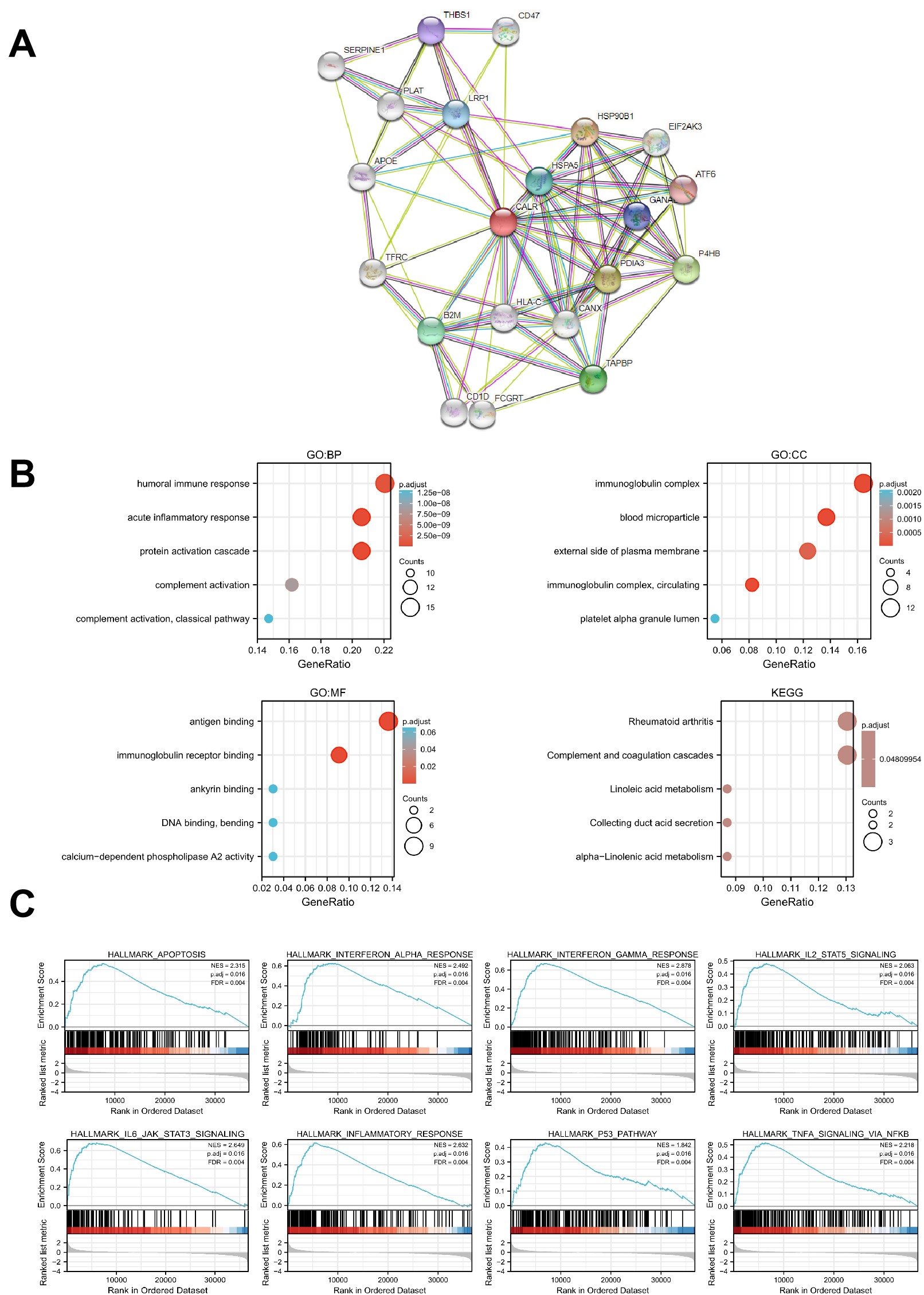

Supplement: Supplementary file 2 [file DataSheet2.ZIP › Figures/Figure 6:Functional and enrichment analysis of CALR in TCGA-KIRC..jpg]

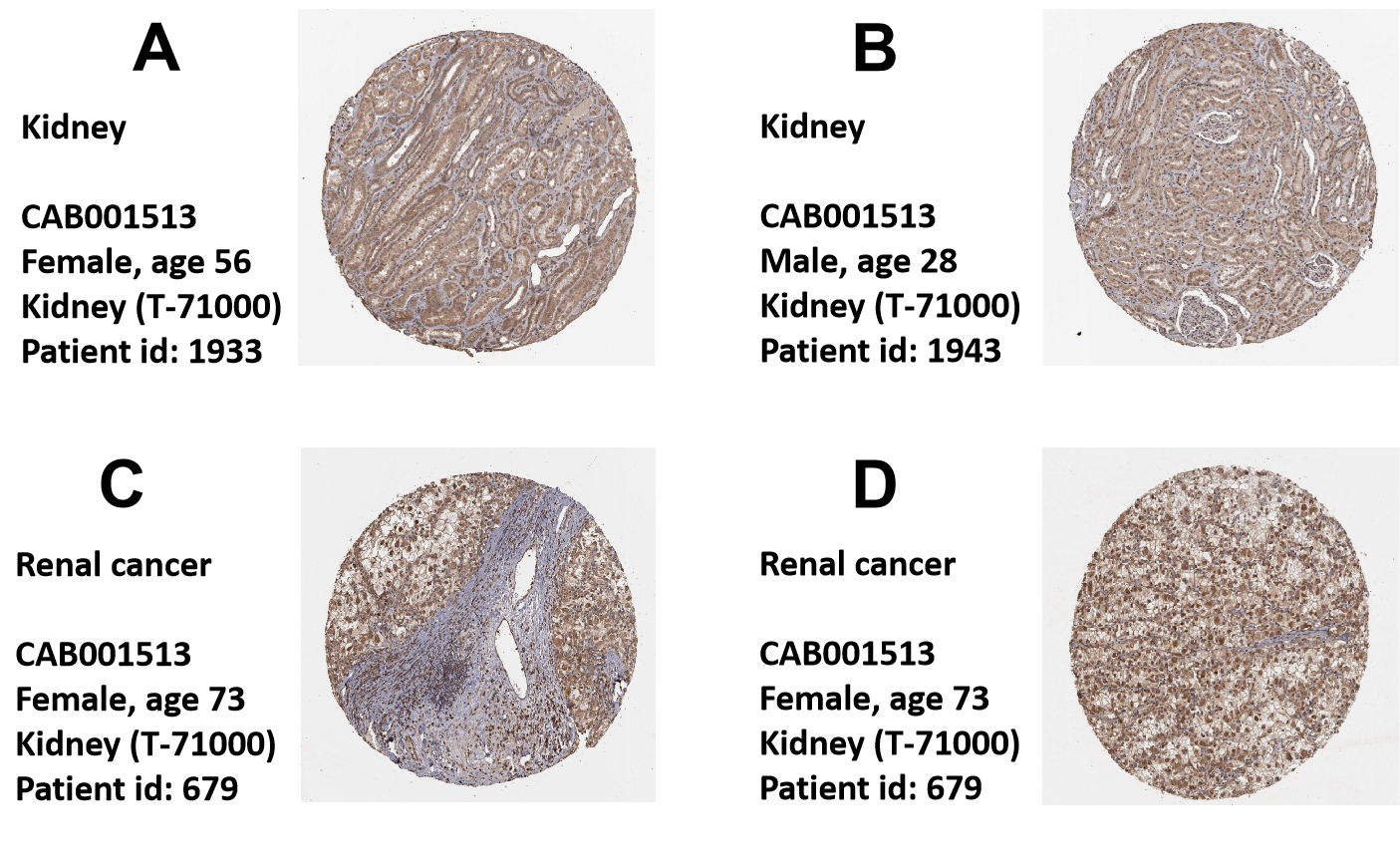

Supplement: Supplementary file 2 [file DataSheet2.ZIP › Figures/Figure 3:Expression of CALR in healthy control and KIRC patient's tissues..tiff]

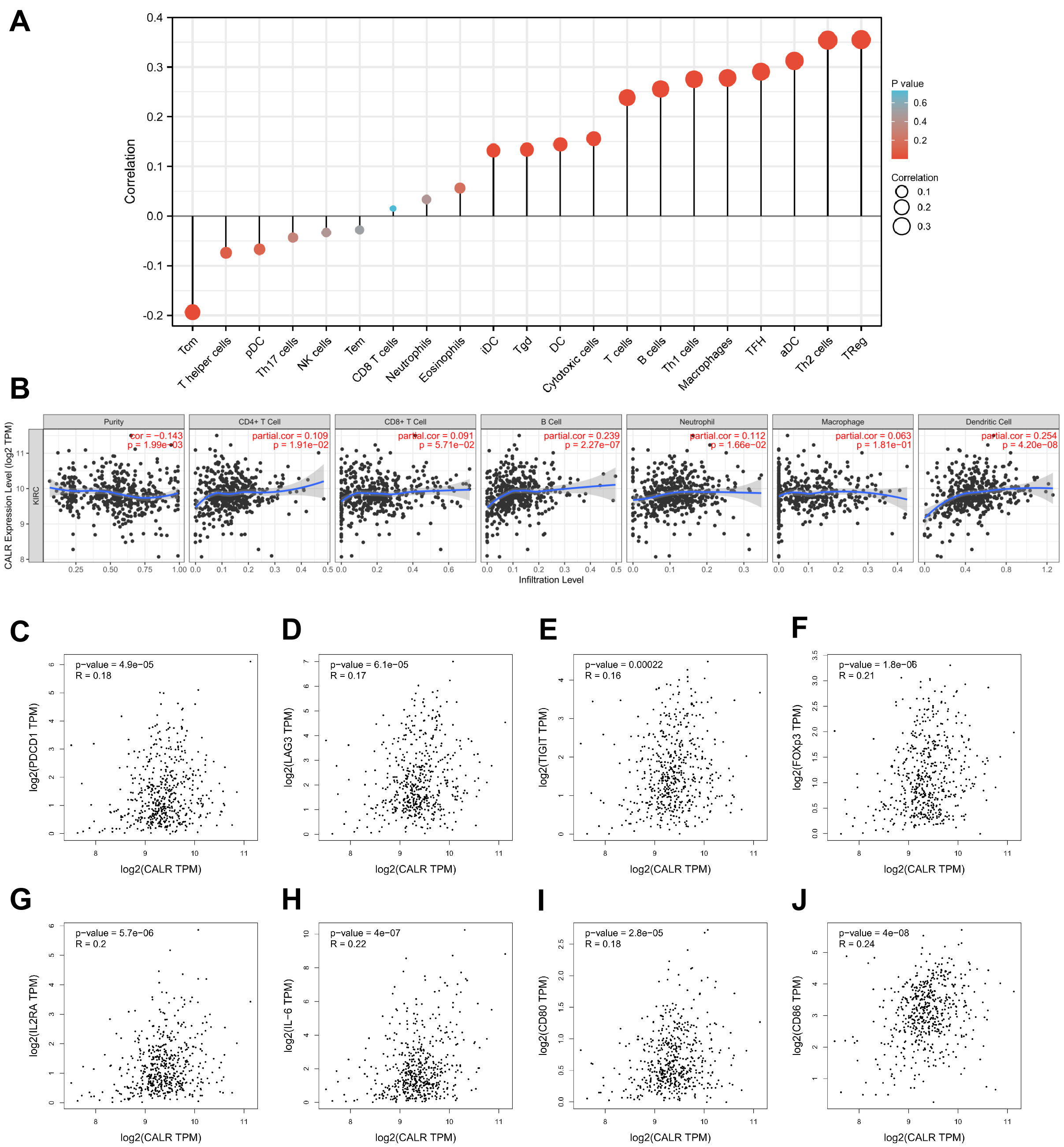

Supplement: Supplementary file 2 [file DataSheet2.ZIP › Figures/Figure 7:Correlation between CALR expression and immune infiltration in TCGA-KIRC..tiff]
